# Transplantation and Noninvasive Longitudinal *In Vivo* Imaging of Parathyroid Cells: A Proof-of-Concept Study

**DOI:** 10.1177/09636897241241995

**Published:** 2024-03-30

**Authors:** Robert Bränström, Pim P. van Krieken, Robin Fröbom, C. Christofer Juhlin, Ivan Shabo, Barbara Leibiger, Ingo B. Leibiger, Per-Olof Berggren, Craig A. Aspinwall

**Affiliations:** 1 Endocrine and Sarcoma Surgery Unit, Department of Molecular Medicine and Surgery, Karolinska Institutet, Stockholm, Sweden; 2 Department of Breast, Endocrine and Sarcoma, Karolinska University Hospital, Stockholm, Sweden; 3 The Rolf Luft Research Center for Diabetes and Endocrinology, Karolinska Institutet, Stockholm, Sweden; 4 Department of Oncology-Pathology, Karolinska Institutet, Stockholm, Sweden; 5 Department of Pathology and Cancer Diagnostics, Karolinska University Hospital, Stockholm, Sweden; 6 Departments of Chemistry & Biochemistry and Biomedical Engineering, and BIO5 Institute, University of Arizona, Tucson, AZ, USA

**Keywords:** parathyroid, eye, transplantation, *in vivo*

## Abstract

The parathyroid cell is a vital regulator of extracellular calcium levels, operating through the secretion of parathyroid hormone (PTH). Despite its importance, the regulation of PTH secretion remains complex and not fully understood, representing a unique interplay between extracellular and intracellular calcium, and hormone secretion. One significant challenge in parathyroid research has been the difficulty in maintaining cells *ex vivo* for in-depth cellular investigations. To address this issue, we introduce a novel platform for parathyroid cell transplantation and noninvasive *in vivo* imaging using the anterior chamber of the eye as a transplantation site. We found that parathyroid adenoma tissue transplanted into the mouse eye engrafted onto the iris, became vascularized, and retained cellular composition. Transplanted animals exhibited elevated PTH levels, indicating a functional graft. With *in vivo* confocal microscopy, we were able to repetitively monitor parathyroid graft morphology and vascularization. In summary, there is a pressing need for new methods to study complex cellular processes in parathyroid cells. Our study provides a novel approach for noninvasive *in vivo* investigations that can be applied to understand parathyroid physiology and pathology under physiological and pathological conditions. This innovative strategy can deepen our knowledge on parathyroid function and disease.

## Introduction

Hyperparathyroidism is a prevalent endocrine disease in Western populations, ranking second only to diabetes. Although primary hyperparathyroidism (pHPT) is often resolved through surgical intervention, renal hyperparathyroidism (rHPT) necessitates pharmacological management, which is frequently insufficient. It is essential to gain a deeper comprehension of the cellular signaling pathways that culminate in parathyroid hormone (PTH) secretion, considering the urgent need for improved treatments.

Hormone secretion from the parathyroid gland is intricately regulated by extracellular free calcium (Ca^2+^) through its interaction with Ca^2+^-sensing receptors (CaSRs) expressed on the parathyroid cell surface^1^. However, studying parathyroid physiology has proved to be notoriously challenging. This is primarily due to the difficulty establishing and maintaining physiologically relevant cell lines in culture^2^. Current knowledge of parathyroid physiology is mostly derived from primary cell cultures, which do not accurately represent physiological processes. Bovine cell cultures, for instance, have lost their Ca^2+^-dependent secretion, likely due to a significant decrease in CaSR^3,4^. A more promising approach has been the cultivation of parathyroid cells from patients with secondary hyperparathyroidism in aggregates (organoids), which have demonstrated up to 5 months of survival^5^. Studies examining the effects of phosphate on PTH secretion in tissue slices, intact glands, and dispersed cells have shown that while tissue slices and intact glands exhibit PTH secretion in response to phosphate stimulation, dispersed cells do not^6^,^7^. Furthermore, *ex vivo* culture may result in loss of critical, yet unknown, paracrine or other interactions that contribute to physiological regulation. As a result, studying parathyroid physiology in a way that mimics the physiological state remains a challenging task.

Approximating the physiological state may also be achieved by parathyroid transplantation and subsequent analysis *in vivo*. A critically needed advance is the ability to investigate signaling in the parathyroid cells after they have been transplanted into an *in vivo* environment. A common method used both clinically and partly in research settings is the autologous intramuscular transplantation of parathyroid tissue. Speier et al.^8,9^ developed a method to study pancreatic islets following their transplantation into the anterior chamber of the mouse eye (ACE), thus providing a minimally invasive transplantation site that allows direct visualization and longitudinal imaging in an *in vivo* setting. In addition, the anterior chamber is regarded as an immune-privileged niche, minimizing immunological response to the transplanted tissue. Spurred by the success of the ACE model for islet research, we hypothesized that this might also be a plausible model for the study of parathyroid tissue and cells. In this work, we demonstrate, for the first time, successful transplantation of parathyroid cells into the ACE and subsequent imaging, providing a much needed capability to enable novel, and important studies of parathyroid cell function.

## Materials and Methods

### Human Samples

Parathyroid tissues were obtained from patients operated on for pHPT. A small piece (<50 mg) of parathyroid adenoma tissue was immediately taken from the surgical ward to the laboratory in sterile tubes containing physiological 0.9% NaCl solution^10^. The tubes were transported to the laboratory on ice. The parathyroid adenoma tissue was dissociated from macroscopic adipose tissue, vessels, and other connective tissue. Control samples were also taken for pathological verification that it was parathyroid tissue. Small pieces, typically between 0.25 and 0.5 mm, of parathyroid tissue were prepared manually under a microscope while immersed in phosphate-buffered saline (PBS) and stored pending transplantation on ice. The entire procedure from the operation theater to the final preparation took, on average, less than 2 h.

### Animals and Transplantation Procedure

The immunodeficient NSG mouse strain was used for these studies^11^. The exact transplantation procedure has been described in detail for pancreatic islets^8,9^. Briefly, recipient animals were anesthetized with isoflurane and positioned under a stereomicroscope. During transplantation, conventional eye drops, or sterile PBS, were used to keep the eye moist. A small hole was made in the cornea with an injection needle to access the anterior chamber. This procedure is performed very carefully so that neither the lens nor iris is damaged. Small pieces of parathyroid were injected with a tailor-made glass needle connected via tubing to a Hamilton syringe. Small injection volumes were used in all experiments: The total volume of parathyroid tissue suspension into the ACE did not exceed a few microliters per eye. After transplantation, each mouse was given a single injection of 0.05–0.1 mg/kg of buprenorphine for postoperative pain relief once and was observed closely until it showed normal and alert behavior. At the time of euthanasia, it was performed by decapitation. In total, 22 NSG mice (6 males and 16 females) were used in the study. Of these, 11 mice were transplanted (three males and eight females), and 11 controls (three males and eight females) were not subjected to any intervention.

### PTH Measurements

Blood samples were collected from the tail vein into heparin tubes and immediately put on ice. Following centrifugation of the samples, supernatant yielding a volume of a minimum of 50 μl was stored at −20°C. For PTH analysis, a Future Diagnostics (Wijchen, the Netherlands) STAT-IntraOperative-Intact-PTH (STAT-IO-I-PTH) immunoassay kit was used according to the manufacturer’s protocol. It is noteworthy that the PTH detection method employed mirrors the clinical approach used at Karolinska University Hospital, relying on cross-reactivity between human and mouse PTH. PTH levels were expressed as pg × ml^−1^.

### Confocal Microscopy

The imaging procedure for parathyroid grafts was essentially performed as previously described for pancreatic islets^8,9^. In brief, mice were anesthetized with isoflurane, placed in a head holder, and photographs of the eye(s) were made with a digital camera attached to a Leica M60 stereomicroscope. To visualize blood vessels, mice received an intravenous injection with 70 or 500 kDa Fluorescein-5-isothiocyanate (FITC)-labeled dextran (Invitrogen) before confocal imaging. Using a Leica SP5 system equipped with 10× and 25× water-immersion objectives, confocal z-stacks of 2 or 4 µm thickness were recorded upon excitation wavelength (λ) at 488 and 633 nm. Volocity Software version 6.2 (PerkinElmer) was used for image display and analysis of blood vessel density.

### Routine Processing of Resected Eye Specimen and Immunohistochemistry

Following euthanasia and enucleation, graft-bearing eyes were directly fixed in formalin. Next, the eyes were oriented for sagittal sectioning by a surgical pathologist and embedded in paraffin using standard clinical procedures at the Department of Pathology and Cancer Diagnostics, Karolinska University Hospital, Stockholm, Sweden. Tissue blocks were cut at 4 mm, mounted on Superfrost Plus slides, and stained with hematoxylin-eosin (H&E) for routine histopathological evaluation. Additional immunohistochemistry was performed using clinical standard procedures and an automated Ventana methodology (Roche Diagnostics, Basel, Switzerland). Antibodies used were anti-human mouse monoclonal PTH (clone MRQ-31, ready-to-use dilution; Roche Diagnostics) and anti-human mouse monoclonal CD34 (clone QBend/10, ready-to-use dilution, Roche Diagnostics). We used anti-human antibodies developed for clinical usage, relying on cross-reactivity between species. Indeed, the gene sequences show 68% and 65% homology between *Homo sapiens* and *Mus musculus* for PTH and CD34, respectively (www.ensembl.org).

### Statistics

PTH levels were compared using the Student’s *t* test, and *P* values less than 0.05 were considered significant, where *n.s.* denotes not significant. Data are expressed as means ± standard deviation (SD).

## Results

### Engraftment of Parathyroid Tissue Visualized by *In Vivo* Confocal Microscopy

**Figure 1.**
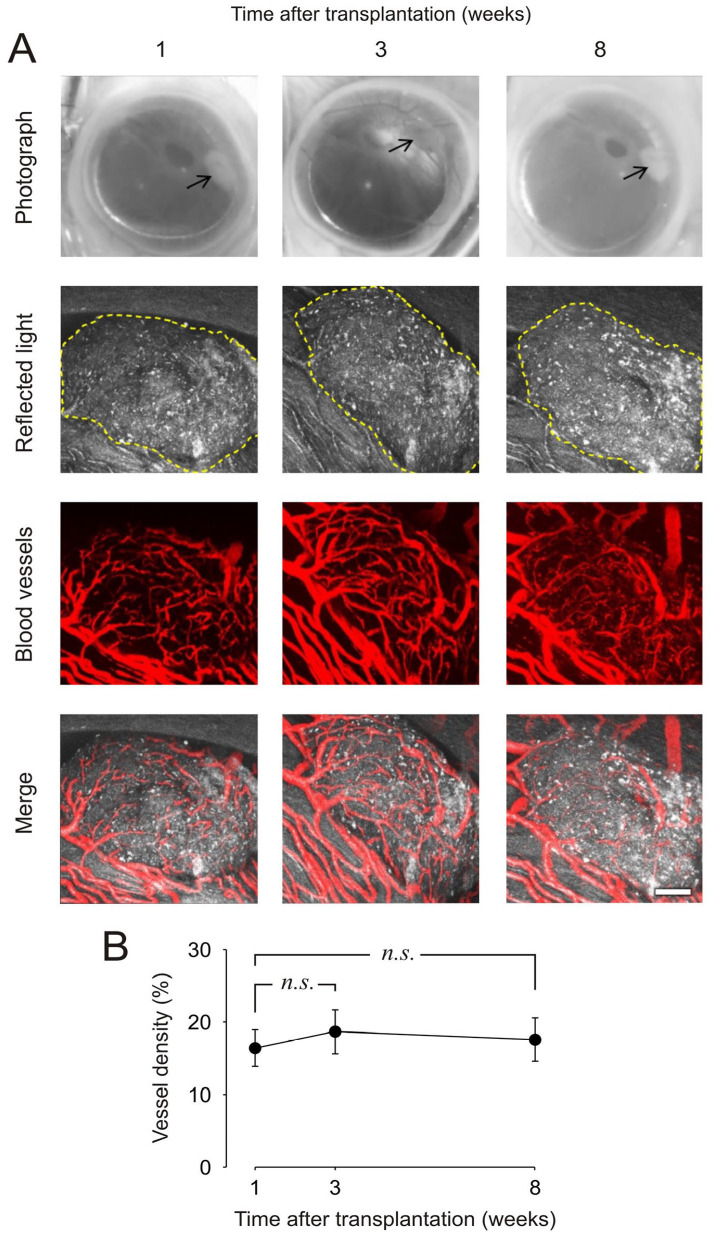
Monitoring of parathyroid tissue engraftment by *in vivo* microscopy. (A) Photos and laser confocal images of a piece of human parathyroid adenoma in the anterior chamber of the eye of a mouse at 1, 3, and 8 weeks after transplantation. Confocal images are presented as maximum projections. A yellow dotted line traces the border of the graft. Blood vessels are visualized by intravenous injection of fluorescent dextran before *in vivo* imaging. Scale bar, 100 μm. (B) Vascular density of parathyroid grafts presented as the area of vessels per tissue area (*n* = 9 grafts in three mice).

Small pieces of human parathyroid gland were successfully injected into the ACE of anesthetized immunodeficient NSG mice. Besides serving as a minimally invasive transplantation site, the ACE enables the monitoring of transplanted tissue over time using *in vivo* confocal microscopy^8,9^. To analyze graft morphology and the revascularization process, we performed reflected light imaging^12^ and collected images of intravenously injected fluorescently labeled dextran at 1, 3, and 8 weeks after transplantation. The photographs and confocal z-stacks of a representative graft displayed in Fig. 1A illustrate that we were able to successfully image parathyroid tissue during engraftment up to 8 weeks posttransplantation. The morphological assessment indicated no significant changes in graft volume over time, demonstrating that human parathyroid tissue survive for at least 8 weeks in the anterior chamber of the eye of an NSG mouse. A relatively dense network of blood vessels was observed at each imaging time point within the parathyroid tissue. Detailed analysis of the dextran-labeled vasculature showed that the maximal blood vessel density was reached 1 week after transplantation (Fig. 1B), suggesting that parathyroid revascularization in the ACE occurs rapidly.

### Functional Studies of PTH Levels in Blood

**Figure 2.**
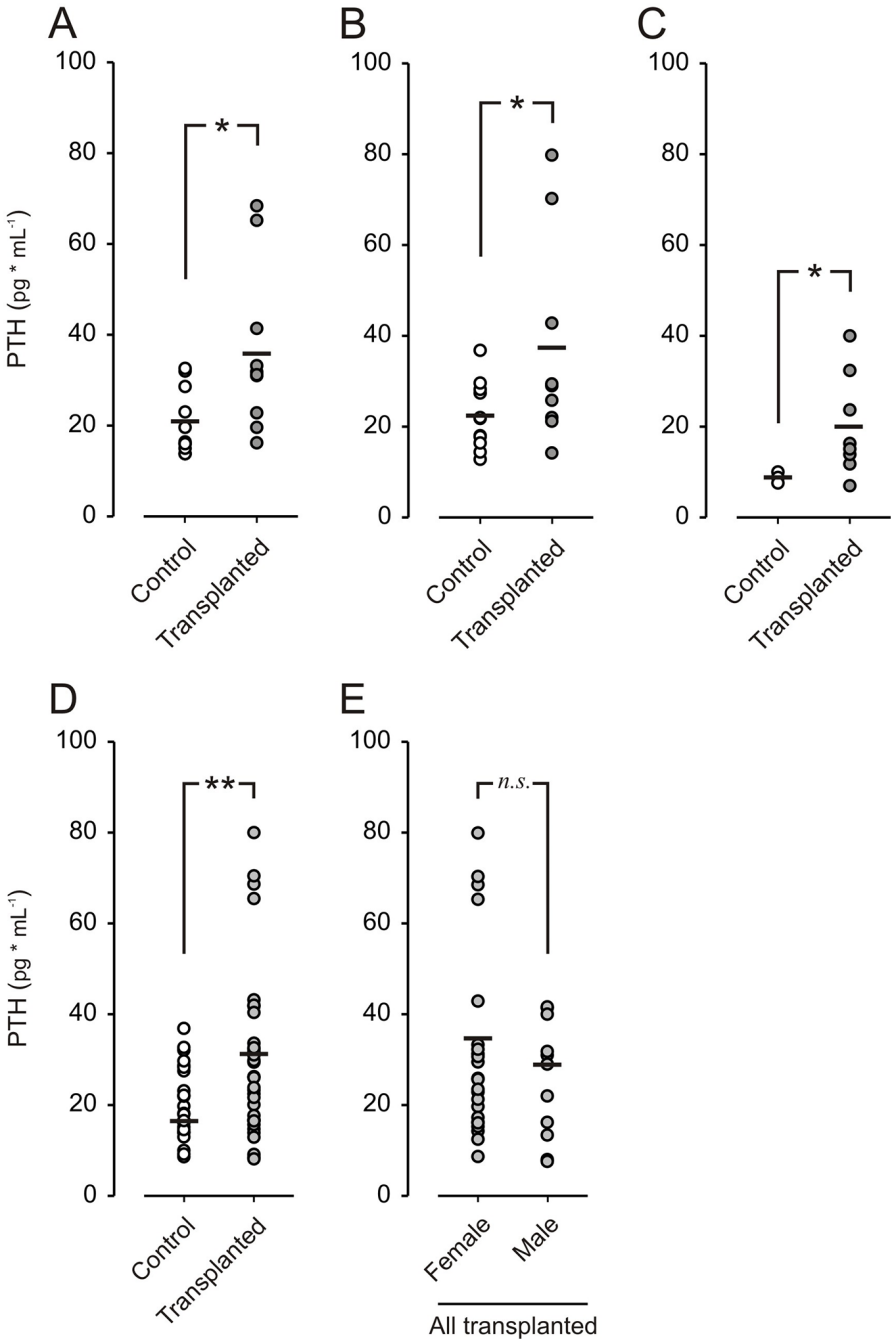
PTH levels in transplanted mice compared to control. (A) PTH from individual control mice (*n* = 11) and transplanted mice (*n* = 10) 3 weeks posttransplantation, in B at 8 weeks posttransplantation (control *n* = 11, transplanted *n* = 9), and in C at 16 weeks posttransplantation (control *n* = 3, transplanted *n* = 8). (D) Summary of all PTH samples in control (*n* = 26) and transplanted (*n* = 36) mice, measured 3, 8, and 16 weeks after transplantation. (E) Summary of all PTH levels from transplanted female mice (*n* = 23) and transplanted males (*n* = 11). * *P* <0.05, ** *P* < 0.01, and *n.s.* denotes not significant.

Blood samples were taken at different intervals (3, 8, and 16 weeks posttransplantation) to measure PTH and evaluate the functional capacity of the graft. In Fig. 2, the PTH levels from different time points were summarized for both transplanted and control mice, with samples pooled according to transplant status. Fig. 2B–D shows the PTH levels of individual control and transplanted mice at 3, 8, and 16 weeks, respectively. Fig. 2D shows a summary of all PTH levels at different time points; both control and transplanted mice and animals that received PTH transplants exhibited a significant increase in serum PTH levels compared with untransplanted controls at all time points, with no observable differences between male and female mice (Fig. 2E).

### Morphological Assessment by Light Microscopy

**Figure 3.**
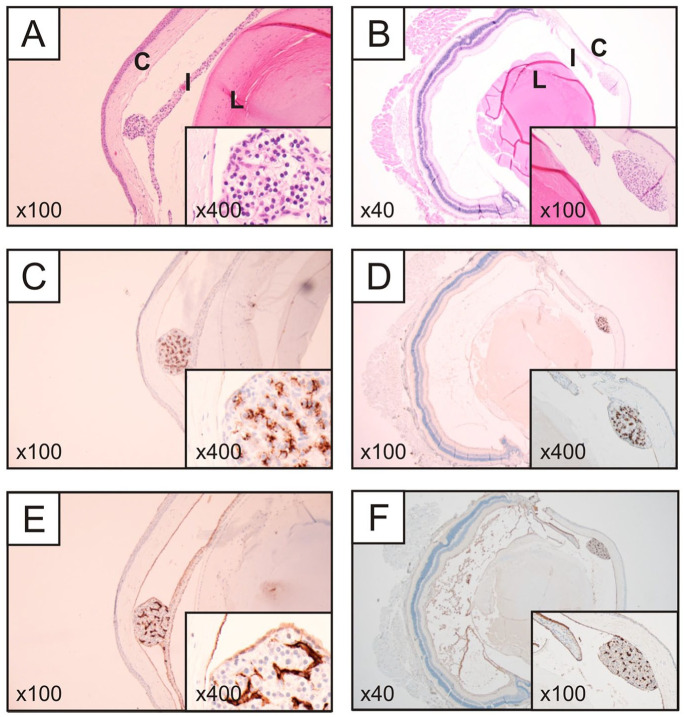
Representative histological and immunohistochemical photomicrographs from a transplanted mouse, left eye (left column) and right eye (right column). (A–B) Routine hematoxylin-eosin stain of a sagittal section. Note the parathyroid cellular mass attached to the iris, with a morphology resembling the chief cells of the normal parathyroid gland (left). (B) The parathyroid cellular mass was attached to the cornea. (C–D) PTH immunostainings reveal intense cytosolic immunoreactivity in most cells, thereby verifying them as parathyroid-derived. (E–F) CD34 immunohistochemistry highlights the endothelial network, suggesting well-preserved vasculature.

Routine H&E stained sections from subsets of the transplanted eyes were investigated by light microscopy by an endocrine pathologist with experience in parathyroid tissue identification and pathological evaluation (Fig. 3). Viable parathyroid chief cell clusters were visualized in all investigated specimens. The grafts were attached to either iris or the cornea and displayed a visible vasculature. Cytosolic PTH staining confirmed the parathyroid origin of the cell clusters (Fig. 3C, D), and CD34 immunohistochemistry identified an endothelial network indicative of well-preserved vasculature (Fig. 3E, F). In control experiments, immunohistochemical staining revealed intense PTH staining in chief cells of the de-identified parathyroid adenoma, with negative immunoreactivity observed in endothelial cells (marked with arrowheads) (Supplemental Fig. 1A). Conversely, control staining of normal colon tissue with CD34 antibody showed specific staining of endothelial cells in the mucosa and submucosa, with the mucosal epithelium remaining negative (marked with arrowheads) (Supplemental Fig. 1B).

## Discussion

Studying parathyroid cell physiology and pathology has been challenging due to the difficulty maintaining primary cultures over extended periods of time, which is typically limited to a couple of days even under optimal conditions^2,10^. Although parathyroid cell lines have been developed in Fabbri et al.^13^ and Sakaguchi et al.,^14^ they are not widely used due to their tendency to lose important physiological properties as they differentiate over time. Moreover, cultured cells and cell lines cannot replicate the complex *in vivo* environment to which cells are exposed, further limiting our understanding of key physiological signaling pathways. These limitations underscore the need for novel approaches to study signaling pathways in parathyroid cells and possibly improve our understanding of their functional relationships. In clinical settings, autologous transplantation of parathyroid tissue is common, with the tissue most commonly transplanted beneath muscle fascia. To address these issues, we sought to explore xenogenic transplantation as a means to directly visualize the parathyroid tissue and its dynamics with respect to engraftment and potential physiological consequences.

We present in this study the first successful xenogeneic transplantation of human parathyroid adenoma tissue into the mouse ACE. This novel transplantation site allows for long-term monitoring of parathyroid survival, vascularization, and functional studies of parathyroid physiology *in vivo*. We employed confocal microscopy coupled with fluorescence biosensors and other techniques to perform functional studies of parathyroid cells. This approach has been previously utilized to study endocrine tissues such as the pancreatic islet with superior results *in vivo*^8,9,11,12^. The ability to study parathyroid cells in this unique environment, along with specific *in vivo* measurement tools and approaches, offers a promising avenue for broader parathyroid cell studies. Such studies may help elucidating intracellular signaling pathways under physiological conditions^15^, and why they are not functioning under pathological conditions. Nevertheless, it is important to acknowledge that the parathyroid tissue employed in this study originated from parathyroid adenomas. Parathyroid adenomas are benign tumors, and while they do secrete PTH, it is essential to recognize that these cells have an affected stimulus-secretion coupling^16,17^.

Following transplantation of human parathyroid grafts into the ACE of NSG mice, fluorescence imaging and histological examination revealed the presence of tissue of parathyroid origin that also expressed endothelial markers indicative of a vascular network (Fig. 3). *In vivo* fluorescence imaging studies at different time points showed quick revascularization within 1 week after transplantation. In addition, the presence of PTH revealed by the histological stain supports the continued production of PTH within the transplanted tissue grafts over an extended time frame following transplantation. Increased serum PTH levels, as seen in Fig. 2, further indicate that the vascular network is sufficient to maintain tissue survival and function. Of note, we were unable to measure serum calcium due to insufficient available blood volume. Continued fluorescence imaging at 3 and 8 weeks showed little additional vascularization (Fig. 1). In comparison, pancreatic islets transplanted into the ACE usually develop dense vasculature within 3–4 weeks. It is known that parathyroid proliferative lesions, such as adenomas, exhibit increased angiogenesis^18^, and also the PTH stimulatory effect of vascular endothelial growth factor (VEGF) in the endothelium^19^ could explain the quicker vascularization. From the imaging studies, and frequent graft observation, no obvious signs of parathyroid cell death, via necrosis or apoptosis, were observed based on the consistent size of the graft after the first week. Importantly, this work demonstrates that parathyroid tissue transplanted into the ACE is suitable for both confocal fluorescence imaging and reflected light imaging, which may open many avenues for future investigations, provided that the tissue grafts are maintained within the 100- to 200-mm limits for dense granule-containing tissues.

The ACE has proven to be a feasible transplantation site, enabling the future study of parathyroid physiology as well as pathology *in vivo*, which has thus far not been possible due to anatomical constraints. The milieu in the ACE is rich in vasculature and oxygen and, at the same time, provides an immune-privileged site initially. Our experiments demonstrate that functional studies on parathyroid tissue can be performed for at least 8 weeks and indicate that the tissue is engrafted with functional vasculature and active PTH secretion within 3 weeks. Our observation that transplanted parathyroid tissue is vascularized as early as 1 week posttransplantation suggests that the graft is well received and survives *in vivo*, and detailed temporal dynamics of early vascularization was not possible due to first time point at 1 week. Microscopic examination revealed no signs of infiltrative immune cells, suggesting that the local inflammation remained low with no evidence of graft-versus-host reaction. Although imaging experiments were terminated after 8 weeks, animal survival was significantly longer with more than half of the animals with PTH xenografts surviving for more than 6 months (with no PTH measurements after 16 weeks), with visual PTH tissue remaining in the ACE for all animals at death.

Total serum PTH analysis supports prolonged maintenance of secretory function (Fig. 2E) from the transplanted parathyroid tissue. Assessing the contribution of the parathyroid graft to whole-body PTH levels in transplanted mice is challenging because the assay used in this study cross-reacts and detects both mouse and human PTH. The similar sequence homology between mice and humans makes a method differentiating them difficult to obtain with antibody-based techniques. However, the PTH was higher in transplanted group that should not be affected by the intervention other than engrafted and functioning parathyroid tissue. The functional capacity of the graft was likely retained as deduced from repeated total serum PTH measurements at different time points. PTH levels were statistically higher in transplanted mice compared with untransplanted controls. We hypothesize that this observation is attributed to the PTH secretion from the graft. PTH secretion exhibits a constitutive component; thus, increased PTH tissue volume should lead to increased serum PTH levels. We note that the increased variability in hormone levels in transplanted animals compared with untransplanted controls may be due to differences in the volume of implanted PTH tissue per animal. Future studies will focus on more controlled implantation compared with this proof-of-concept study. While the function of PTH tissue xenografts is supported by the imaging and secretion data, further studies are warranted to optimize and more thoroughly characterize the transplantation procedure to enable future functional studies of the PTH xenografts.

To conclude, growing parathyroid tissue *in vitro* has proven to be quite challenging, with cells and tissue often surviving only a few days even under optimal conditions. This proof-of-concept study demonstrates the feasibility of transplanting human parathyroid tissue into the mouse ACE. The functional readouts on PTH levels suggest the tissue’s physiological integrity, while imaging studies reveal rapid revascularization and engraftment. The transplantation of parathyroid tissue into the mouse’s eye should be primarily seen as an experimental platform for *in vivo* experiments. In future research, prioritizing the integration of fluorescence-based biosensors can enhance the investigation of parathyroid intracellular signaling pathways under conditions that closely resemble the physiological environment. The field has struggled with extended culture periods for cells, and this approach provides a promising way to overcome this limitation.

## Supplemental Material

sj-jpg-1-cll-10.1177_09636897241241995 – Supplemental material for Transplantation and Noninvasive Longitudinal In Vivo Imaging of Parathyroid Cells: A Proof-of-Concept Study Supplemental material, sj-jpg-1-cll-10.1177_09636897241241995 for Transplantation and Noninvasive Longitudinal In Vivo Imaging of Parathyroid Cells: A Proof-of-Concept Study by Robert Bränström, Pim P. van Krieken, Robin Fröbom, C. Christofer Juhlin, Ivan Shabo, Barbara Leibiger, Ingo B. Leibiger, Per-Olof Berggren and Craig A. Aspinwall in Cell Transplantation
