# Supplementary figures and images for: Transplantation and Noninvasive Longitudinal In Vivo Imaging of Parathyroid Cells: A Proof-of-Concept Study
Source: Cell Transplant. 2024 Mar 30;33:09636897241241995. doi: 10.1177/09636897241241995 (PMC10981846; doi:10.1177/09636897241241995)

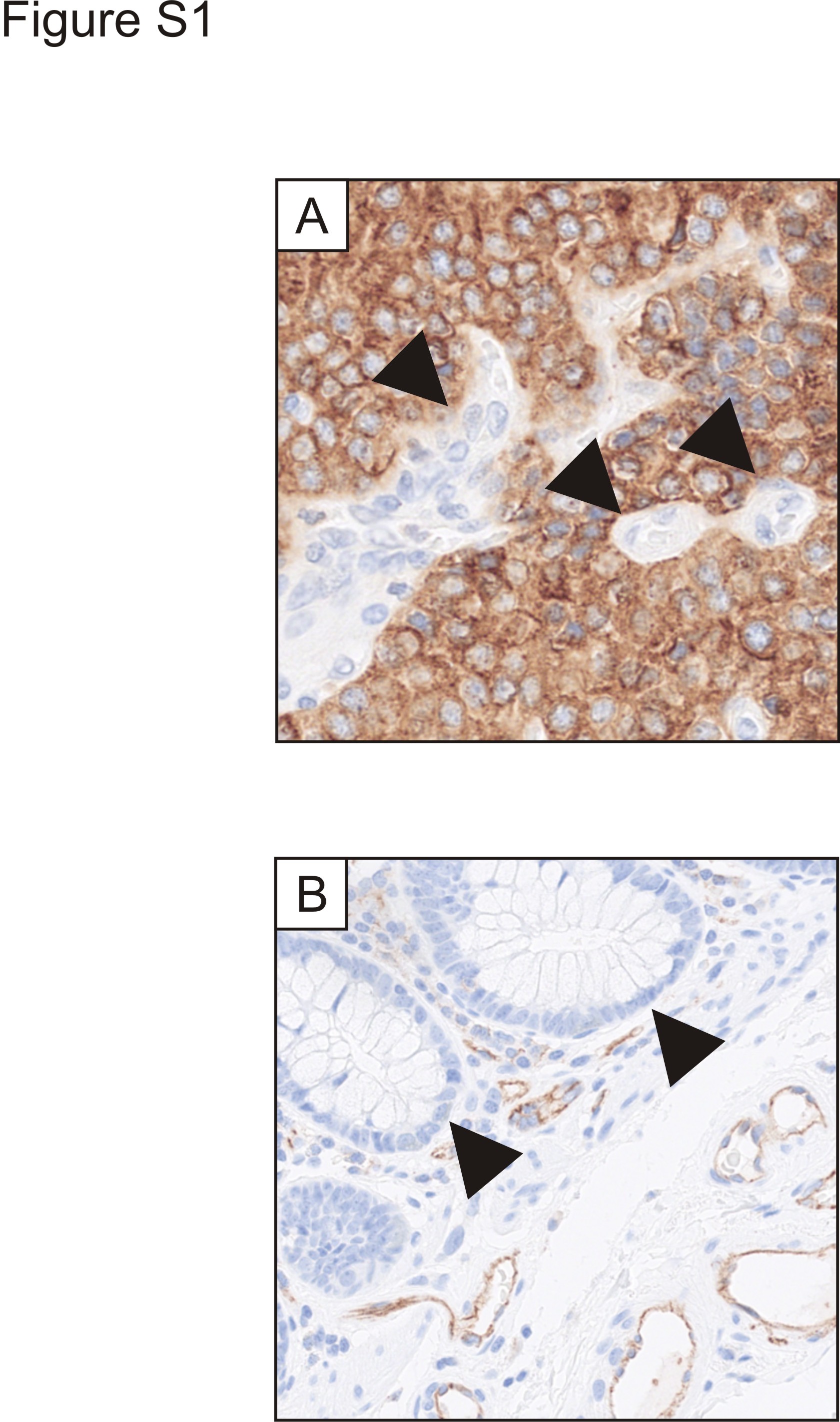

Supplement: sj-jpg-1-cll-10.1177_09636897241241995 – Supplemental material for Transplantation and Noninvasive Longitudinal In Vivo Imaging of Parathyroid Cells: A Proof-of-Concept Study [file sj-jpg-1-cll-10.1177_09636897241241995.jpg]
